# IAA-producing plant growth promoting rhizobacteria from *Ceanothus velutinus* enhance cutting propagation efficiency and Arabidopsis biomass

**DOI:** 10.3389/fpls.2024.1374877

**Published:** 2024-05-14

**Authors:** Jyothsna Ganesh, Katherine Hewitt, Ananta Raj Devkota, Ty Wilson, Amita Kaundal

**Affiliations:** Plants, Soils, and Climate, College of Agriculture and Applied Sciences, Utah State University, Logan, UT, United States

**Keywords:** rhizobacteria, microbiome, *Pseudomonas*, phosphate solubilization, siderophore production, native plants, PGPR - plant growth-promoting rhizobacteria

## Abstract

Climate-induced drought impacts plant growth and development. Recurring droughts increase the demand for water for food production and landscaping. Native plants in the Intermountain West region of the US are of keen interest in low water use landscaping as they are acclimatized to dry and cold environments. These native plants do very well at their native locations but are difficult to propagate in landscape. One of the possible reasons is the lack of associated microbiome in the landscaping. Microbiome in the soil contributes to soil health and impacts plant growth and development. Here, we used the bulk soil from the native plant *Ceanothus velutinus* (snowbrush ceanothus) as inoculant to enhance its propagation. Snowbrush ceanothus is an ornamental plant for low-water landscaping that is hard to propagate asexually. Using 50% native bulk soil as inoculant in the potting mix significantly improved the survival rate of the cuttings compared to no-treated cuttings. Twenty-four plant growth-promoting rhizobacteria (PGPR) producing indole acetic acid (IAA) were isolated from the rhizosphere and roots of the survived snowbrush. Seventeen isolates had more than 10µg/mL of IAA were shortlisted and tested for seven different plant growth-promoting (PGP) traits; 76% showed nitrogen-fixing ability on Norris Glucose Nitrogen free media,70% showed phosphate solubilization activity, 76% showed siderophore production, 36% showed protease activity, 94% showed ACC deaminase activity on DF-ACC media, 76% produced catalase and all of isolates produced ammonia. Eight of seventeen isolates, CK-6, CK-22, CK-41, CK-44, CK-47, CK-50, CK-53, and CK-55, showed an increase in shoot biomass in *Arabidopsis thaliana*. Seven out of eight isolates were identified as *Pseudomonas*, except CK-55, identified as *Sphingobium* based on 16S rRNA gene sequencing. The shortlisted isolates are being tested on different grain and vegetable crops to mitigate drought stress and promote plant growth.

## 1 Introduction

Global warming induces climate change, increases global temperature, elevates atmospheric carbon dioxide, and changes precipitation patterns. It is the biggest threat to ecosystems and biodiversity (Allen et al., 2010). These changes increase drought, soil salinity, flooding, weeds, and pests, directly impacting crops and agriculture’s growth and development (FAO, 2009). Drought is expected to be the leading cause of reduced crop production by 2050 (Vinocur and Altman, 2005). After Nevada, Utah is considered to be the second driest state in the United States (Utah Department of Public Safety). On top of that, anthropogenic abuses and an increased frequency of extreme weather negatively affect soil health. Chemical fertilizers are used extensively to enhance crop production. However, adding these to the soil affects soil health and crop production through sanitization, water pollution, organic matter, and high costs (Olanrewaju et al., 2017; Pahalvi et al., 2021).

Landscaping, the major consumer of irrigation water, is an essential section of agriculture prone to adverse effects caused by environmental stresses. Recent studies using crop water demand and green water availability projections under changing climates have shown that we can adapt agriculture to green water scarcity through various management strategies and have the potential to promote global food security (He and Rosa, 2023). Promoting and using native plants in urban landscaping is in demand in arid urban areas in China (Liu et al., 2024). Native plants that grow in their natural habitat with little human intervention are promising in low-water landscapes (Rupp and Wheaton, 2014). The degraded lands, wildlife habitats, and wetlands have been restored by using these native plants (Hooper et al., 2005). The U.S. Intermountain West is rich in drought-tolerant woody and herbaceous native plants, which can symbiotically interact with actinobacteria *Frankia* and fix atmospheric nitrogen (Benson, 1982; Kratsch and Graves, 2004; Kratsch, 2011). Most of Utah is a wilderness rich in native plants and endemic species. Native plants are of keen interest to the Green Industry as they have traits adapted to their native environments, including drought and salt tolerance. Desert and dry-land native plant species are adapted to arid and semiarid conditions because of their deep root systems and are insect and fungi-resistant. They need less harsh chemicals such as fertilizers and pesticides that may harm natural environments and soil. Some native plants are recommended for low-water landscaping (Mee et al., 2003; Kratsch, 2011; Rosentreter et al., 2017). Although some native plants do very well in the wild, they are challenging to grow in a nursery or landscape environment. One possible reason for this could be a lack of natural symbioses with soil microorganisms in the landscape. For example, *Ceanothus velutinus* (snowbrush ceanothus) is challenging to propagate for nursery production and is recognized as an actinorhizal plant. They have a wide range of distribution from 2133- 2743 m elevation. The seeds of this plant show 80-90% germination if adequately collected and treated (Rupp and Wheaton, 2014). The roots of snowbrush ceanothus cutting are susceptible to rotting, and therefore, it is challenging to grow from cuttings in a propagation mix (Rupp and Wheaton, 2014). One possible reason for this failure to propagate could be the lack of native soil microbiome in the landscape media.

Soil is known as the most diverse and complex microbial ecosystem on the land (Crowther et al., 2019). The soil microbe-plant interactions are complex and are significant in plants’ growth and development (Lugtenberg and Kamilova, 2009). One gram of soil contains around 10 billion diverse microbes in and around the plant roots (Yadav et al., 2017). Plants secrete high or low molecular weight organic compounds as root exudates in the soil that attract the selective bacteria and colonize (Walker et al., 2003). A Study has shown that *Pseudomonas stutzeri* NRCB010 induces certain exudates from the roots of Tomato, which enhances the microbe’s colonization in the rhizosphere (Zhang et al., 2023). The microbe in the bulk soil around the roots acts as an inoculum and directs microbes to colonize the rhizosphere and roots (Bulgarelli et al., 2015). The microbes inhabiting the rhizosphere or roots, which can be bacteria, fungi, and viruses, exhibit several plant growth-promoting traits like phosphate solubilization, Indole-3-acetic acid (IAA), Nitrogen fixation, siderophore, catalase, ammonia production, ACC deaminase and protease activity and help plant’s growth and development (Mohanty et al., 2021).

Many PGPR are known to produce phytohormones like auxin, cytokinin, and several volatile organic compounds (VOC) that promote plant growth and help plants mitigate various biotic and abiotic stresses (Orozco-Mosqueda et al., 2023). They have also been shown to increase shoot length and diameter (Karlidag et al., 2007; Karakurt and Aslantas, 2010). IAA is a prominent member of the phytohormone auxin in plants and controls many plant growth activities, from root architecture to fruit and embryo development, phototropism, geotropism, apical dominance, leaf formation, and abscission (Srivastava, 2002). The tryptophan-dependent pathway is the best-understood pathway of IAA production in plants and microbes (Zhao, 2010). Natural and synthetic auxins such as IAA, IBA, naphthaleneacetic acid (NAA), and 2,4-dichlorophenoxyacetic acid (2,4-D) are in regular use for plant cells, tissue, and organ cultures to elicit specific morphogenetic responses, such as callusing and rooting However, IAA is very unstable; still, it is used for plant tissue cultures (Nissen and Sutter, 1990). Bacteria can improve overall plant health by producing IAA that can induce root architecture alterations that enhance water and nutrient uptake (Glick, 2012; Pii et al., 2015; Etesami et al., 2015b). In addition, bacterial IAA helps plants mitigate abiotic stresses such as drought, salinity, and heavy metal toxicity (Etesami and Glick, 2024). Besides, IAA PGPR has shown the ability for nutrient uptake, such as the ability to solubilize phosphate, nitrogen fixation, and siderophore, protease, and catalase production (Kumar et al., 2022). Besides all these plant growth-promoting traits in microbes, 1-aminocyclopropane-1-carboxylic acid (ACC) deaminase enzyme activity in several bacteria is helpful for plants to deal with ethylene stress. ACC is the precursor for the stress hormone ethylene in plants. ACC deaminase cleaves the ACC into ammonia and α-ketobutyrate and inhibits ethylene production. The microorganism with this enzyme activity in the rhizosphere reduces plant ethylene levels, inhibits its detrimental effects, and protects against biotic and abiotic stresses (Glick, 2014). Several plant growth-promoting bacteria have been isolated from the rhizosphere of pineapple from various stress-inducing habitats (Ratnaningsih et al., 2023).

Snowbrush ceanothus rhizosphere has a rich microbiome containing several plant-promoting bacteria that showed several plant growth-promoting characteristics such as IAA, catalase and siderophore production, phosphate solubilization, and the ability to fix nitrogen (Ganesh et al., 2022). The majority of rhizobacteria belonged to *Pseudomonas* and *Streptomyces*. However, there is a variety of PGPR belonging to *Bacillus*, *Peribacillus*, *Variovorax*, *Xenophilus*, *Brevundimonas*, *Pantoea*, *Ancylobactor*, and *Priestia* exhibiting various plant growth-promoting traits, including IAA, siderophore, and catalase production (Ganesh et al., 2022). We hypothesize that the native soil of snowbrush ceanothus harbors many growth-promoting bacteria that produce IAA, which can help the propagation of snowbrush ceanothus in greenhouse conditions and colonize the rhizosphere. In this study, we investigated the effect of bulk soil of the snowbrush ceanothus plants from Logan Canyon as inoculum on the cutting propagation of the snowbrush ceanothus in the greenhouse. We also isolated the IAA-producing rhizobacteria from the surviving cuttings and assessed them seven other PGP traits. Subsequently, we tested their ability to promote plant growth in Arabidopsis.

## 2 Materials and methods

### 2.1 Effect of native soil on the growth of snowbrush ceanothus

#### 2.1.1 Sample collection

We collected bulk soil samples of snowbrush ceanothus plants from the Tony Grove region of Logan Canyon, Utah – elevation 1950m AMSL (Above Mean Sea Level) (41°52’34” N 111°34’20” W, as described elsewhere (Ganesh et al., 2022). The bulk soil samples from the top 30 cm layer around the plant were collected in one gallon Ziplock bags, kept on ice, transported to the lab, and stored at 4°C. The six cm of growing tips of the shoots were collected from the same plant in June and July 2020. The cuttings were wrapped in wet tissue papers and stored in Ziplock bags on ice, brought to the greenhouse, and kept on ice until treated.

#### 2.1.2 Effect of native soil on the rooting and survival of the cuttings of snowbrush ceanothus

The cuttings were cleaned in a 1% Zerotol solution and wound at the bottom. The wounded stems were dipped in 3,000 mg/L indole butyric acid (a rooting hormone Hormodin 2). For the control set, the treated cuttings were placed in peat moss and perlite (1:4 v/v) soil mixture (Paudel et al., 2022). In the second set, 50% of the soil mix was replaced with bulk soil from the snowbrush ceanothus plants in Tony Grove in Logan Canyon to make it 50% of native soil inoculum to observe the effect of microbiome in the native soil on the rooting. The third set of experiments replaced all the soil mixtures with bulk soil. All three cuttings were placed on a mist bench. The temperature of the bottom pads was set to 23°C (Propagation Mat, Grower’s Nursery Supply) with a misting of 60 VPD units with VPD mist controller (Phytotronics, Earth City, Montana). After eight weeks, the cuttings were observed for rooting. The rooted cuttings were transplanted to 3.8 L pots (peat moss (75%), vermiculite (13%), rice husks (12%), wetting agent (AguaGro G, and hydrated lime- CaCO_3_) (Paudel et al., 2022). After ten months of transplantation of cuttings, the survival rate was recorded.

### 2.2 Isolation of bacteria from the rhizosphere of survived cuttings

As described elsewhere, the roots and rhizosphere were collected from the survived cuttings from the above experiment (McPherson et al., 2018). The roots were collected with sterilized scissors in pre-sterilized 50mL tubes containing phosphate buffer [6.33 g/L NaH_2_PO_4_, 8.5 g/L Na_2_HPO_4_ anhydrous, pH = 6.5, 200 mL/L Silwet-L77]. The roots in tubes were shaken on a rotary shaker, and rhizosphere soil was separated from the roots. The roots were removed from the tubes, and tubes were centrifuged at, for five minutes; the supernatant was discarded, and the rhizosphere soil pellets were washed with phosphate buffer without surfactant by centrifugation at 3,000g for five minutes and stored at 4°C. The rhizosphere soil from the five samples was pooled together. One gram of rhizosphere pellet of pooled samples was resuspended in 9.5 mL of sterilized water to a 10:95 ratio of pellet to water. The rhizosphere soil suspension was serially diluted up to 10^-5^. 100 μl of the last three dilutions were spread plated onto the five bacterial growth media viz., ¼ - Strength Nutrient Agar, ¼ - Strength Tryptic Soy Agar, Yeast Mannitol Agar, Minimal M9 Media, and Actinomycete Isolation Agar at 28°C for 3-5 days. After five days, based on morphological characteristics, the unique colonies were purified by the streak plating. The bacterial isolation was done three times. The final isolates were stored as glycerol stocks at -80°C (Ganesh et al., 2022).

### 2.3 Screening of indole acetic acid producing isolates

All isolates were tested for IAA production by colorimetric assay (Sarker and Al-Rashid, 2013). Individual isolates were grown in 5mL LB Broth containing 0.1% tryptophan at 28°C for 72 hours, control having no inoculum. The culture was centrifuged at 3,000g for 10 minutes to collect supernatant. Salkowski reagent was prepared by mixing 2 mL of 0.5 M FeCl_3_ in 49 mL of water and carefully adding 49 mL of 70% perchloric acid. 2 mL of Salkowski was combined with 1 mL of bacterial supernatant and incubated for 25 minutes at room temperature for color development. IAA-producing isolates develop a pink color. Spectramax Microplate reader read a change in absorbance at 530 nm for 300µL of the mixture. An IAA standard curve of 0, 5, 10, 20, 50, and 100 µg/mL of IAA concentration was plotted, and IAA concentration was measured using the equation (**Supplementary Figure 2A**) (Ganesh et al., 2022). Triplicates were taken for each isolate, and the experiment was repeated twice.

### 2.4 Characterization of isolates for plant growth-promoting activities

**Figure 1:**
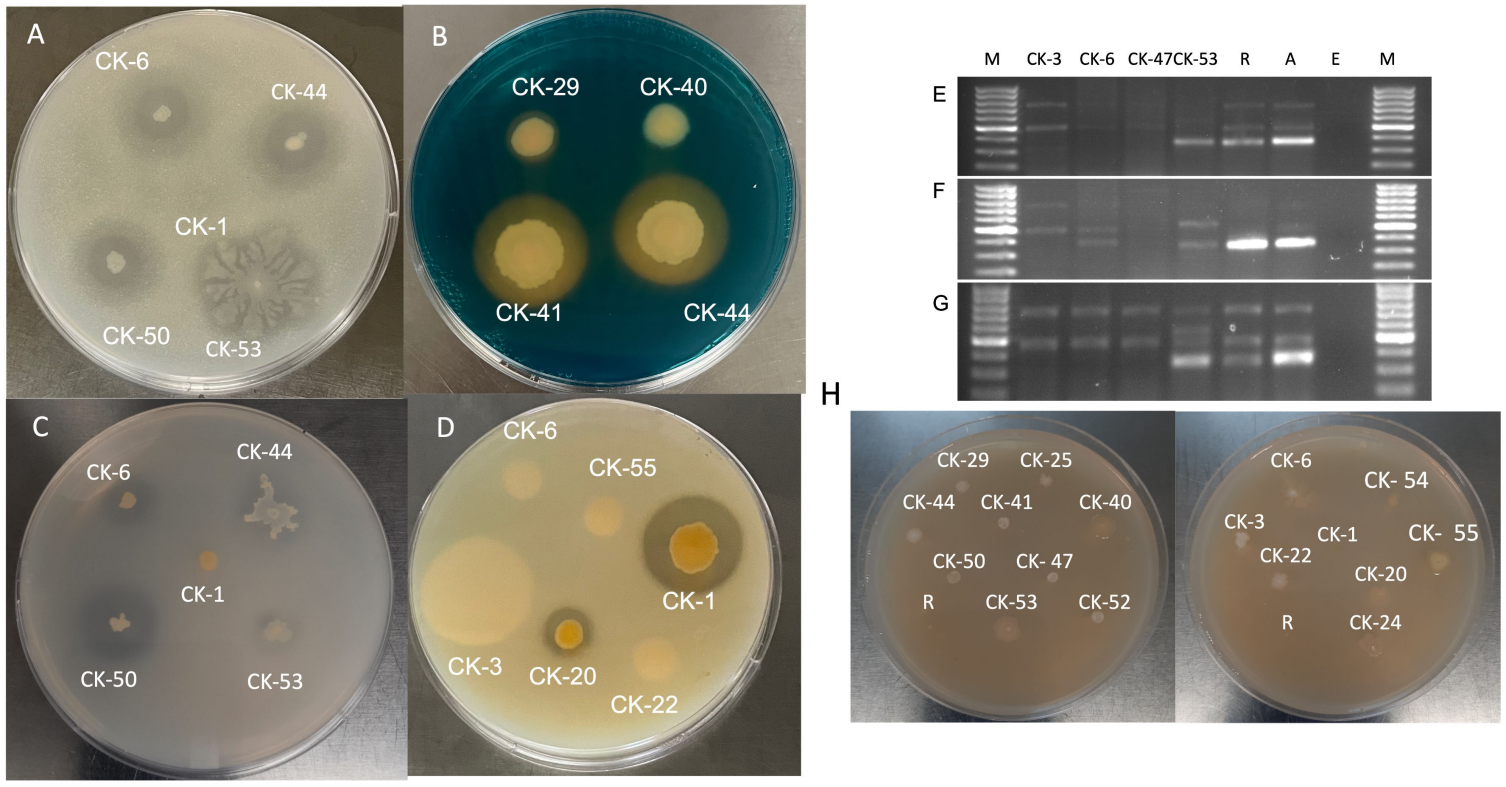
**(A)** Nitrogen fixation on Norris Glucose Nitrogen Free media. **(B)** Siderophore production on CAS media. **(C)** Phosphate solubilization on Pikovskaya agar. **(D)** Protease activity on Skim Milk Agar. Amplification of *nif*H*^+^(*Fe subunit of nitrogenase gene. **(E)** at 55°C, **(F)** 59°C, and **(G)** 62°C. M- 100 base pair ladder, R- *Rhizobium leguminosarum* C6, A-*Azotobactor chroococcum* ATCC 9043, and E– *E. coli* DH 5⍺. **(H)** Isolate growth on DF-ACC media.

Norris’s Glucose Nitrogen-Free Media (Himedia) was used to screen isolates for N-fixing ability. Isolates were spot-plated on the media and incubated for 3-5 days at 28°C. The clear halo formation around the colony showed the nitrogen fixation ability of the isolate (Wafula et al., 2020; Gaete et al., 2022). *Rhizobium leguminosarum* C6 and *Azotobactor chroococcum* ATCC 9043 were used as positive controls. The positive isolates on media were screened for a 393bp fragment of the Fe protein subunit of the *nif* H^+^ (Nitrogenase gene). The bacterial genomic DNA was extracted from each isolate, and 50ng DNA was used with PolF- (5′ TGC GAY CCS AAR GCB GAC TC 3′), PolR- (5′ ATS GCC ATC ATY TCR CCG GA 3′) primers with Dream Taq polymerase at three different annealing temperatures (55°C, 59°C, and 62°C **Figures 1E, F**) as described (Poly et al., 2001; Fernandes Júnior et al., 2013; Ganesh et al., 2022). Each isolate was tested to produce ammonia using Nessler’s reagent with slight modification (Kavamura et al., 2013). Each bacterial isolate was grown in 5 mL Peptone water for 72 hours, and the supernatant was collected by centrifugation for 10 minutes at 3,000g. 20µL of Nessler’s reagent was added to 200µL of supernatant in a 96-well microtiter plate and incubated for 30 min for brown to yellow color development. The absorbance was read at 520 nm on a specromax plate reader. A standard curve using 0, 25, 50, 100,150, and 200 µg/mL of ammonium carbonate was prepared, and ammonia production was calculated using an equation (**Supplementary Figure 2B**). *E. coli* DH5⍺ was used as a positive control.

CAS (Chrome Azurol S) agar media (Millipore SIGMA) was used to test all isolates for siderophore production. The CAS reagent was made according to described elsewhere (Schwyn and Neilands, 1987). The CAS agar was media prepared, as described by Arora and Verma (2017). The bacterial isolates were spot-plated on the CAS media plates and incubated at 28°C for 3-5 days. A clear halo around the bacterial growth on blue color media indicates siderophore production. *P. chlororaphis* O6 was used as the positive control. The experiment was repeated three times. Pikovskaya agar media (Himedia) was used to test isolates for phosphate solubilizing activity (Pikovskaya, 1948). The bacterial isolates were spot-plated on the PS media plates and incubated for 3-5 days at 28°C. The clear halo around the bacterial growth indicates the phosphate solubilizing capability of the isolate. The diameter for bacterial growth and halo was measured. *B. magaterium (*ATCC 14511) was used as the positive control. The phosphate solubilizing index (PSI) was calculated as a ratio of halo diameter over colony diameter (Nacoon et al., 2022). Hydrogen peroxide was used to test for catalase activity in all the isolates (Pakpour and Horhan, 2021). The single bacterial colony was picked on the glass slide, and 1-2 drops of H_2_O_2_ were poured over the bacteria. The production of bubbles indicates catalase activity by bacteria. The experiment was repeated three times.

All bacterial isolates were assessed to possess ACC deaminase enzyme activity on Dworkin and Foster (DF) minimal salt media [DF salt per L, 4.0 g KH_2_PO_4_, 6.0 g Na_2_HPO_4_, 0.2 g MgSO_4_ - 7H_2_O, 2.0 g glucose, 2.0 g gluconic acid and 2.0 g citric acid; Trace elements - (1 mg FeSO4 - 7H_2_O, 10 µg H_3_BO_3_, 11.19 µg MnSO_4_ - H_2_O, 124.6 µg ZnSO_4_ - 7H_2_O, 78.22 µg CuSO_4_ - 5H_2_O, and 10 µg MoO_3_), at pH 7.2] supplemented with 3 mM ACC as sole nitrogen source (Penrose and Glick, 2003). The isolates were spot inoculated in triplicate on DF media plates with ACC and incubated for 3-5 days at 28°C. The isolates that were able to grow on DF media showed the ACC deaminase activity to catabolize ACC as a Nitrogen source (Gupta and Pandey, 2019). The screening was done three times. Skim milk agar media (Skim milk Powder 28.0g, Tryptone 5.0g, yeast extract 2.5g, Dextrose 1.0 g, and Agar 15.0g for 1 liter) was used to test all isolates for protease activity (Nayab et al., 2015). The isolates were spot-plated on the Skim milk agar plates and incubated at 28°C for 3 days. A clear halo around the bacterial growth indicated the protease activity of the isolates. *B. megaterium (*ATCC 14511) and *B. subtilis* were used as positive controls.

### 2.5 Identification of isolates by 16S rRNA gene sequencing

The 1.4 KB, 16S rRNA gene fragment was amplified from each isolate using 27F (V1 region- 5′-AGAGTTTGATCCTGGCTCAG-3′) and 1492R (V9 region- 5′-TACGGYTACCT TGTTACGACTT-3′) set of primers with DreamTaq DNA polymerase as described elsewhere (Ganesh et al., 2022). The bacterial suspension was prepared for each isolate by resuspending a single colony in 20 µL of sterilized water and boiling at 98°C for 10 minutes. 2µL of the bacterial suspension was used as a template for PCR. The PCR products were sequenced and were searched against the 16S rRNA gene database on BLAST (Basic Local Alignment Search Tool). The sequences for each isolate were deposited to GenBank on NCBI, and accession numbers are in **Table 1**.

**Table 1:** Isolated producing more than 10µg/mL of Indole Acetic Acid (IAA).

S. No.	Code	Blast	IAA (µg/mL)	Accession Number
1	CK-1	*Chryseobacterium* sp.	27.69^±1.73^	OR795730
2	CK-3	*Pseudomonas* sp.	27.76^±0.58^	OR795731
3	CK-6	*Pseudomonas* sp.	10.95^±0.02^	OR795732
4	CK-20	*Massilia* sp.	32.09^±0.30^	OR795733
5	CK-22	*Pseudomonas* sp.	23.78^±0.36^	OR795735
6	CK-24	*Bacillus* sp.	16.09^±0.72^	OR795734
7	CK-25	*Pseudomonas* sp.	11.79^±0.12^	OR795736
8	CK-29	*Pseudomonas* sp.	23.04^±0.67^	OR795737
9	CK-40	*Klebsiella* sp.	41.06^±0.40^	OR795738
10	CK-41	*Pseudomonas* sp.	18.68^±0.59^	OR795739
11	CK-44	*Pseudomonas* sp.	28.79^±0.54^	OR795740
12	CK-47	*Pseudomonas* sp.	19.17^±0.20^	OR795741
13	CK-50	*Pseudomonas* sp.	10.31^±0.23^	OR795742
14	CK-52	*Pseudomonas* sp.	29.11^±0.42^	OR795743
15	CK-53	*Pseudomonas* sp.	19.53^±0.18^	OR795744
16	CK-54	*Bacillus* sp.	18.25^±0.11^	OR795745
17	CK-55	*Sphingobium* sp.	15.43^±0.12^	OR795746

### 2.6 Plant growth promotion on *Arabidopsis thaliana*

The seventeen isolates based on more than 10µg/mL of IAA production were tested on the model plant *Arabidopsis thaliana* Col-0 for growth promotion. *A. thaliana* seeds were germinated LC1 metro mix (SUNGRO,WA, USA). The Arabidopsis plants were maintained in the growth chamber at 22°C/18°C day/night, a photoperiod of 8-h/16-h day/night, a light intensity of 160 μmol m^−2^ s^−1^, and relative humidity was ~50.

Bacterial inoculum was prepared as described elsewhere (Tefera and Vidal, 2009; Fan et al., 2020). Bacterial isolates from glycerol stocks were streaked on ¼-Strength Nutrient Agar and incubated at 28°C for 24 hours. An individual colony for each isolate was inoculated into 5 mL LB broth in a 13 mL culture tube and incubated at 28°C/180 rpm for 24 hours. The colonies were subcultured for 48 hours in 100 mL of LB broth. The inoculum has been prepared in 1/8- Strength Murashige and Skoog nutrient at OD_600_- 1.0.

At the four-leaf stage, the one-week-old seedlings were transferred into individual pots on LC1 mix and inoculated with 5mL solution by pouring on the base of the plant. Plants were inoculated with 5 mL 1/8 – Strength MS as controls. One week later, the plants were inoculated twice after one-week intervals for a total of three inoculations. Plants were randomized within each tray and watered approximately every 4 days. After two months of the first treatments, the plants were harvested, and each plant’s fresh and dry shoot weight was measured. Five biological replicates were used for data collection. An analysis of variance (ANOVA) was done, and the Tukey-Kramer method for multiplicity at ɑ = 0.05 was used to calculate the significant difference.

## 3 Results

### 3.1 Native soil promotes propagation of snowbrush ceanothus

**Figure 2:**
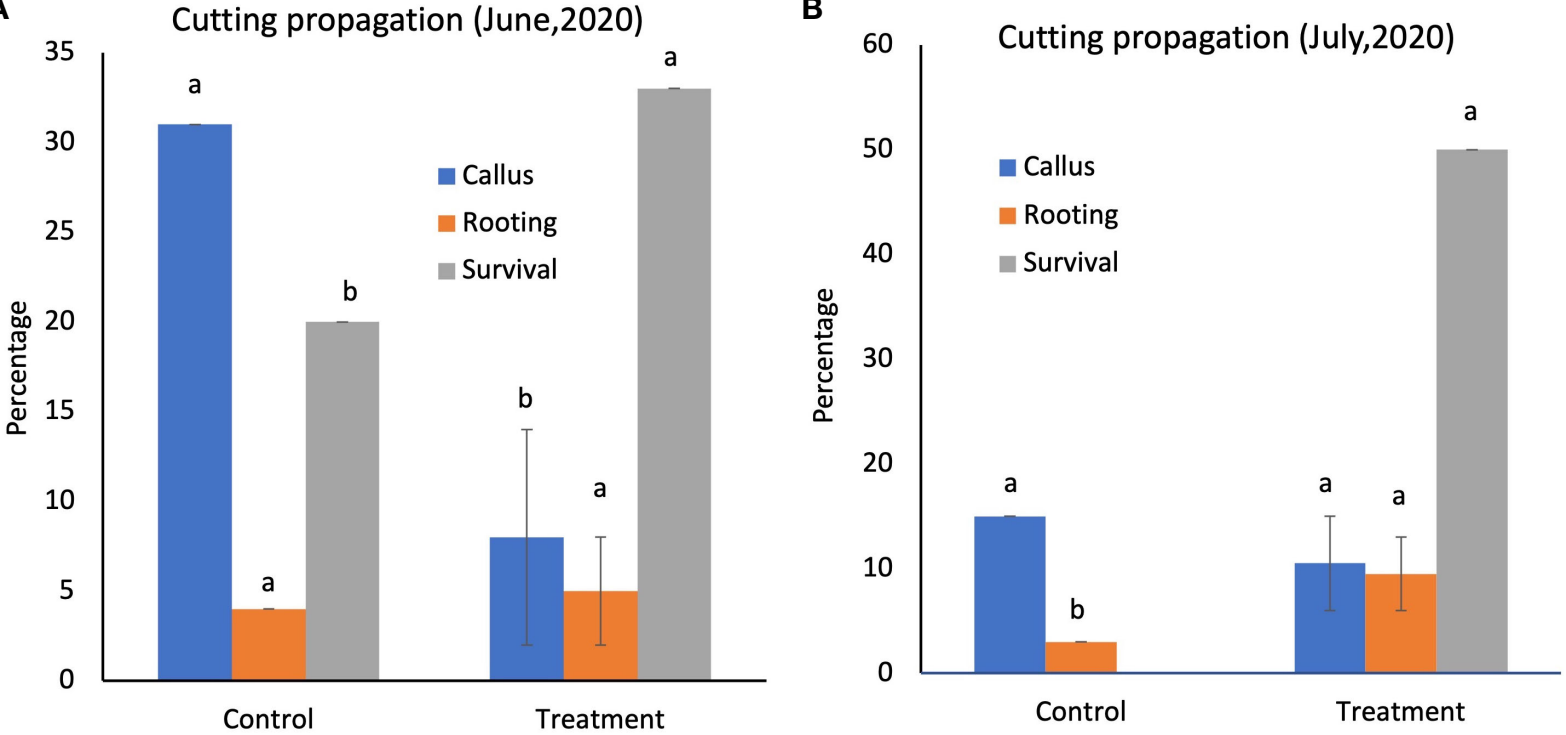
**(A)** Cutting propagation in June 2020, the survival percentage of the cuttings treated with 50% of the native soil from 1950 (33%), which was significantly higher than the control (20%). **(B)** Cutting propagation in July 2020, the survival rate percentage was significantly higher (50%) in native soil-treated cuttings than in the control cuttings (0%). Different alphabets show significant differences in the Tukey-Kramer method for multiplicity at α < 0.05. Modified with permission from Ganesh, 2021.

The snowbrush ceanothus cuttings treated with native soil were observed for callus formation and rooting after 6-8 weeks. The 100% native soil experiment did not yield successful rooting as it retained too much moisture. The rooted cuttings from 50% native soil inoculation were transplanted into the 1-gallon pot containing 75% peat moss, 13% vermiculite, and 12% rice husks. The native soil-treated cutting showed denser and longer roots than the control (**Supplementary Figure 1**). The callusing, rooting, and survival percentages were recorded for the June and July experiments (**Figure 2**). The June experiment results showed that the callus formation was significantly greater (α<0.05) in the control (31%) than in the 50% native soil treatment from 1950 (3%). However, the rooting and survival of the treated cuttings (5 and 33%) were more significant (α<0.05) than the control (4 and 20%) (**Figure 2A**). It showed that the rooting and survival rate of native soil treated cutting was higher even when they had less callusing than control cuttings. Even in the July experiment, the callus formation was higher in the control (15%) than in the 59% native soil treatment (10%). However, the rooting and survival percentage was significantly higher (α<0.05) in 50% native soil-treated cuttings (9.5 and 50%) than in the control cuttings (3 and 0%) (**Figure 2B**). These results indicate that the inoculation of native soil to propagate the mix helps the snowbrush ceanothus cuttings with more rooting and survival.

### 3.2 IAA-producing plant growth-promoting bacteria

Fifty-five bacteria were isolated from the rhizosphere of the cuttings on ¼ NA, TSA, and YMA media. Based on morphological characteristics, twenty-seven unique colonies out of 55 were selected and tested for indole acetic acid production (**Supplementary Table 1**). Except for two isolates, CK – 7 and CK – 11, all isolates showed more than 1 µg/mL of IAA production (**Supplementary Table 2**). Seventeen isolates CK-1, CK-3, CK-6, CK-20, CK-22, CK-24, CK-25, CK-29, CK-40, CK-41, CK-44, CK-47, CK-50, CK-52, CK-53, CK-54, and CK-55 showed IAA production of more than 10 µg/mL (**Table 1**). CK-40 produced the highest amount of IAA which was 41.06^±0.40^ µg/mL followed by CK-20, which produced 32.09^±0.30^ µg/mL. All seventeen isolates were sequenced for the 16S rRNA region. 50% of isolates identified as *Pseudomonas*. The remaining isolates, CK24 and CK-54, were *Bacillus*, CK-1 was *Chryseobacterium*, CK-20 was *Massilia*, CK-40 was *Klebsiella*, and CK-55 was *Sphingobium* sp.

### 3.3 Isolates also exhibit several other plant growth-promoting traits

#### 3.3.1 Ability to fix nitrogen

76% of the isolates (13/17) CK-3, CK-6, CK-22, CK-25, CK-29, CK-40, CK-41, CK-44, CK-47, CK-50, CK-52, CK-53, and CK-55, showed ability to fix nitrogen when screened using Norris Glucose Nitrogen Media (**Table 2**). These isolates formed a halo around the bacterial colony growth (**Figure 1A**). However, only two isolates, CK-6 and CK-53, amplified the 393bp fragment of the nitrogenase gene, and both were identified as *Pseudomonas*. The fragment was amplified in the CK-6 at 55°C; in CK-53, it was amplified at all three annealing temperatures (**Figures 1E–G**). Most of the isolates for N- fixation CK-3, CK-6, CK-22, CK-25, CK-29, CK-41, CK-44, CK-47, CK-50, CK-52, and CK-53 were identified *Pseudomonas*. However, CK-40 was *Klebsiella*, and CK-55 was *Sphingobium* sp.

**Table 2:** Characterization of shortlisted isolates for plant growth promoting traits.

S. No.	Code	IAA (µg/mL)	PSI	NH_3_ (µg/mL)	NF		SPI	ACC	PA	Cat
					Media	*nif*H^+^				
1	CK-1	27.69^±1.73^	0	40.98^±4.10^	–	–	+++	–	+++	–
2	CK-3	27.76^±0.58^	0	104.05^±2.87^	++	–	+	++	–	++
3	CK-6	10.95^±0.02^	2.4^±0.23^	56.84^±5.35^	++	+	++	+++	–	++
4	CK-20	32.09^±0.30^	0	11.79^±3.74^	–	–	–	++	++	–
5	CK-22	23.78^±0.36^	2.40^±0.39^	17.34^±2.86^	+++	–	++	++	–	+++
6	CK-24	16.09^±0.72^	0	64.06^±14.30^	–	–	–	++	+	–
7	CK-25	11.79^±0.12^	1.41^±0.24^	17.90^±4.76^	+++	–	+	+	–	+++
8	CK-29	23.04^±0.67^	1.82^±0.02^	47.25^±0.74^	+++	–	+	+	–	+++
9	CK-40	41.06^±0.40^	2.93^±0.23^	80.71^±10.66^	+++	–	–	+++	–	+++
10	CK-41	18.68^±0.59^	2.11^±0.26^	18.41^±3.96^	+++	–	++	+	–	+++
11	CK-44	28.79^±0.54^	2.01^±0.13^	50.32^±5.83^	+++	–	++	+	–	+++
12	CK-47	19.17^±0.20^	1.96^±0.12^	72.72^±2.92^	+++	–	++	+	–	+++
13	CK-50	10.31^±0.23^	2.43^±0.03^	100.69^±8.39^	+++	–	++	+	++	+++
14	CK-52	29.11^±0.42^	2.17^±0.12^	16.90^±3.07^	++	–	+	+	–	++
15	CK-53	19.53^±0.18^	2.05^±0.05^	85.98^±4.81^	+	+	++	+++*	+	+
16	CK-54	18.25^±0.11^	1.48^±0.12^	105.35^±2.87^	–	–	–	+	+	–
17	CK-55	15.43^±0.12^	0	77.50^±14.12^	+	–	–	+++	–	+

**‘-’** negative/absent, ‘+’ mild positive/present, ‘++’ moderately positive, ‘+++’ strongly positive, *- growing extensively. SP, Siderophore production; PS, Phosphate solubilization; IAA, Indole Acetic Acid production (µg/mL); ACC, ACC deaminase activity; PA, Protease activity; Cat, Catalase production; NF, Nitrogen Fixation; *nif*H^+^(Fe subunit of nitrogenase gene), NH_3_- Ammonia production. ^±^ Standard Error for triplicates.

#### 3.3.2 Ammonia production

All the bacterial isolates produced some amount of ammonia. However, eleven isolates (64%) produced more than 50µg/mL of ammonia, and three isolates, CK-3, CK-50, and CK-54, produced more than 100µg/mL of ammonia (**Table 2**). *E. coli* DH5⍺ produced 176µg/mL of ammonia.

#### 3.3.3 Siderophore production

70% of (12/17) isolates CK-1, CK-3, CK-6, CK-22, CK-25, CK-29, CK-41, CK-44, CK-47, CK-50, CK-52, and CK-53 produced halos on CAS media and showed siderophore production. The isolate CK-1 showed the largest halos (**Table 2**; **Figure 1B**). Except for CK-1, all other isolates were identified as *Pseudomonas*. CK-1 was identified as *Chryseobacterium*.

#### 3.3.4 Phosphate solubilization

70% of (12/17) Isolates CK-6, CK-22, CK-25, CK-29, CK-40, CK-41, CK-44, CK-47, CK-50, CK-52, CK-53, and CK-54 can solubilize phosphate when tested on Pikovskaya agar media and produced halos. CK-40, identified as *Klebsiella*, showed the largest PSI of 2.93^±0.23^, followed by CK-50, CK-6, and CK-22,which were *Pseudomonas* with PSIs of 2.43^±0.03^, 2.40^±0.23^, and 2.40^±0.39^, respectively (**Table 2**; **Figure 1C**). Most isolates were *Pseudomonas* except CK-40 and CK-54, which were *Klebsiella* and *Bacillus*, respectively.

#### 3.3.5 Catalase production

Thirteen out of seventeen isolates CK-3, CK-6, CK-22, CK-25, CK-29, CK-40, CK-41, CK-44, CK-47, CK-50, CK-52, CK-53, and CK-55, produce catalase. 53% of the microbes produced an abundance of catalase, which created bubbles from 5-9mm when 1 drop of hydrogen peroxide was added to a single colony, denoted by +++ (Strongly Positive) **Table 1**. 41% were able to produce a 2-5mm bubble formation (++) (Moderately positive). The remaining 6% produced bubbles in a circle that spanned 0.1-2mm in diameter (+) mild positive (**Table 2**). Most catalase-producing isolates were *Pseudomonas* except CK-40, *Klebsiella*, and CK-55, *Sphingobium.*

#### 3.3.6 ACC deaminase activity

All the isolates except CK-1, identified as *Chryseobacterium*, could grow on DF media and showed ACC deaminase activity. Eight isolates CK- 25, CK-29, CK-41, CK-44, CK-47, CK-50, CK-52, and CK-54 grew mildly on DF media after five days of incubation. Four isolates, CK-3, CK-20, CK-22, and CK-24, grew moderately, while four isolates CK-6, CK-40, CK-53, and CK-55, grew very well on the DF media and used ACC as a source of Nitrogen (**Table 2**; **Figure 1H**).

#### 3.3.7 Protease activity

Six out of 17 (36%) isolates CK-1, CK-20, CK-24, CK-50, CK-53, and CK-54 showed protease activity when screened on skim milk agar (**Table 2**; **Figure 1D**). CK-1 and CK-53 showed maximum activity, with CK-53 growing extensively on skim milk agar. Three isolates, CK-24, CK-50, and CK-53 were identified as *Pseudomonas*. The three isolates CK-1, CK-20, and CK-54 were identified as *Chryseobacterium, Massilia*, and *Bacillus.*

**Figure 3:**
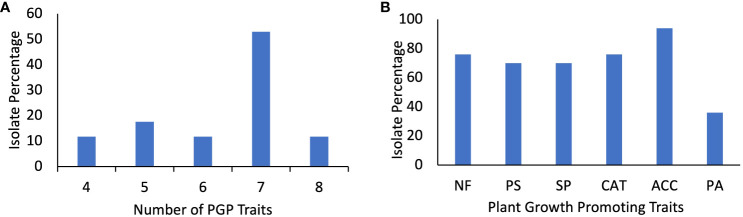
**(A)** Percentage of the isolates for the number of plant growth-promoting traits. **(B)** Percentage of isolates showing Nitrogen Fixation (NF) on Norris Glucose Nitrogen Free media, Phosphate solubilization (PS), Siderophore production (SP), Catalase production (CAT), ACC deaminase activity on DF-ACC media, and protease activity (PA).

All seventeen isolates tested positive for four or more PGP traits. 47% (8/17) of isolates CK-6, CK-22, CK-25. CK-29, CK-41, CK-44, CK-47, and CK-52 showed seven PGP traits, while two isolates, CK-50 and CK-53, tested positive for all eight PGP traits, three isolates CK-3, CK-54, and CK-55 showed five PGP traits. CK-60 showed six PGP traits (**Figure 3A**; **Table 2**).

### 3.4 Isolates enhance the biomass of *A. thaliana*

**Figure 4:**
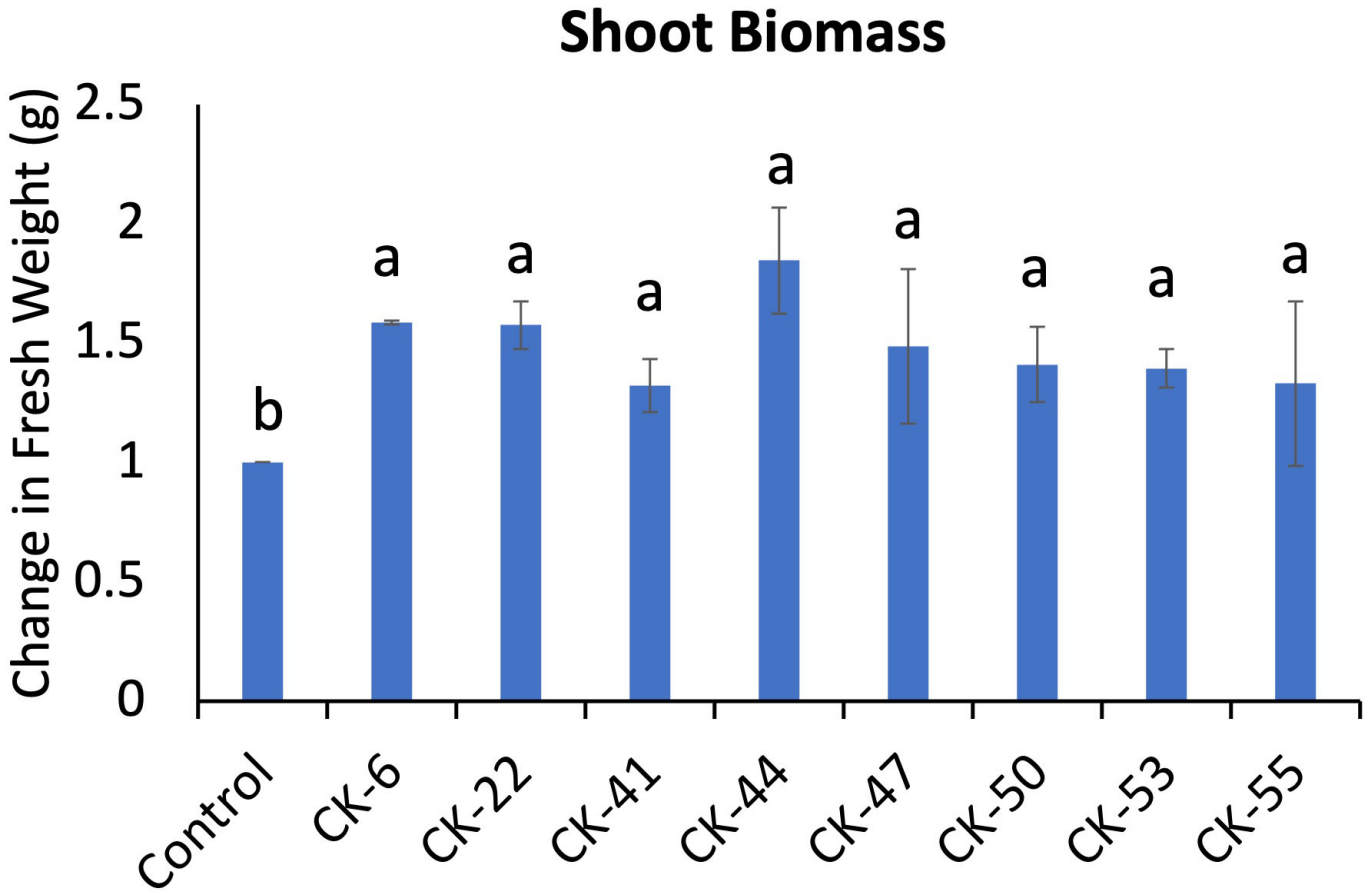
Eight bacterial isolates from the rhizosphere of snowbrush ceanothus cuttings showed a significant increase in biomass in *Arabidopsis thaliana*. Different alphabets show significant differences in the Tukey-Kramer method for multiplicity at α < 0.05.

Eight bacterial isolates, CK-6, CK-22, CK41, CK-44, CK-47, CK-50, CK-53, and CK-55, out of 17 tested on the *A. thaliana*, showed a significant increase in shoot biomass compared to non-treated plants (**Figure 4**). Seven isolates promoting plant growth belonged to *Pseudomonas*, while one isolates, CK-55, was identified as *Sphingobium* sp.

## 4 Discussion

Snowbrush ceanothus is a challenging native plant to propagate in landscape (Rupp and Wheaton, 2014). A recent study showed several PGPR in the rhizosphere of native soil-treated greenhouse-grown ceanothus plants (Ganesh et al., 2022). This study revealed the inoculation of native soil to the propagation mix with Hormodin-2 to snowbrush ceanothus cuttings in the greenhouse showed visually longer and denser roots and a higher survival rate of 33% in June and 50% in July (**Supplementary Figure 1** and **Figure 2A**). In soybeans, a study showed that inoculation of soybean seeds with a consortium of indigenous microbes from the adult healthy plants from two different managed soils benefited the seedling growth (May et al., 2023). In another study, the native soil from different crops, such as maize wheat, ryegrass, and sweet potato, was used to inoculate rice plants. This inoculation led to isolating wheat and maize-derived plant growth bacteria, significantly promoting rice plant growth (Habibi et al., 2014).

Various studies reported that PGPR promotes a plant’s root growth by producing indole acetic acid (IAA), an auxin known to regulate root growth and architecture (Vacheron et al., 2013; Grover et al., 2021). We isolated the IAA-producing bacterial isolates from the survived cuttings in the next step, and all isolates except for two isolates, CK-7 and CK-11, produced IAA more than 1 µg/mL. A rice seedling study upon inoculation with rhizosphere and endophytic isolates from rice plant roots reported that the “IAA may be the first PGP trait” for isolation of PGPB (Etesami et al., 2015a). Another study isolated the IAA-producing bacteria from banana, cotton, maize, and wheat rhizosphere. In pot experiments, these isolates showed significant growth promotion in wheat seedlings (Mohite, 2013).

All isolates except CK-1 could grow on DF media, which has ACC as a nitrogen source and may possess ACC deaminase activity. (**Figure 3B**; **Table 2**). Four isolates, CK-6, CK-40, CK-53, and CK-55, grow exclusively on DF media, showing that they possess ACC deaminase activity and can grow on ACC as a nitrogen source. CK-6 and CK-53 were identified as *Pseudomonas*, CK-40 is *Klebsiella* sp., and CK-55 is *Sphingobium* sp. However, four isolates, CK-3, CK-20, CK-22, and CK-24, grow moderately on the DF media. CK-24 was identified as *Bacillus* sp., CK-20 was identified as *Massilia* sp., and CK-3 and CK-22, were identified as *Pseudomonas* sp. The eight isolates grow mildly on DF media (**Figure 3B**). *Klebsiella* strain IG 3 was isolated from the wheat rhizosphere, exhibiting ACC deaminase activity and imparting salt tolerance in oat seedlings (Sapre et al., 2018). ACC deaminase-producing *Pseudomonas* were isolated from the rhizosphere soil of grapevine (*Vitis vinifera* L.) in arid regions of China. Most of these isolates were identified as *Pseudomonas* sp. One of the isolates, *P. corrugata*, weakens the drought-induced growth inhibition in grapevines (Duan et al., 2021). A study reported four ACC deaminase-producing *P. fluorescence* strains improved sweet corn productivity under limited water availability (Zarei et al., 2020). *B. mojavensis* PRN2 from the pea nodules exhibit ACC deaminase activity (Maheshwari et al., 2020). Similarly, three salt-tolerant isolates, *B. subtilis* (NBRI 28B), *B. subtilis* (NBRI 33 N), and *B. safensis* (NBRI 12 M), are reported to promote plant growth and modulate ethylene metabolism to impart salt tolerance in maize (Misra and Chauhan, 2020). Further characterization of these isolates for ACC deaminase activity and drought tolerance can lead to the development of bio stimulants.

One isolate, CK-1, belongs to *Chryseobacterium* sp. and showed extreme protease activity on skim milk agar along with siderophore, ammonia, and IAA production. *Chryseobacterium* soil isolates are known to have several plant-promoting traits, such as IAA, HCN, and antifungal production, and ACC deaminase activities make them useful in crop production. Besides, many species of *Chryseobacterium* have characteristics of degradation of aromatic compounds and play an essential role in industrial use (Nishioka et al., 2016). *Chryseobacterium* isolates from the metal-contaminated site can produce IAA, HCN, and ammonia and promote maize growth in greenhouse experiments (Marques et al., 2010). Similarly, *Chryseobacterium* isolates are known to deamidate proteins, produce proteases, and be used as bioremediation/biodegradation agents (Yamaguchi and Yokoe, 2000; Wang et al., 2008).

Isolate CK-20 was identified as *Massilia* sp. This isolate tested positive for IAA, ammonia production, ACC deaminase, and protease activity. *Massilia* sp. produce many components, such as synthesizing violacein, a natural antibiotic that is effective on malaria, diarrhea, and even tumors, and polyhydroxyalkanoate (PHAs), a kind of natural polymer biomaterial (Agematu et al., 2011; Han et al., 2014). Several *Massilia* sp. have been reported to act as a bioremediation agent and degrade toxic gas components like benzene, toluene, ethylbenzene, and xylene isomers in air and soil contaminants such as phenanthrene and chloroacetamide herbicides (Lou et al., 2016; Lee et al., 2017; Gu et al., 2021; Son et al., 2021). *Massilia* bacteria can also promote plant growth and increase the survival rate of plants in harsh environments. When *Massilia* was applied along with arbuscular mycorrhizal fungi, it alleviated the salt stress on plant growth, root colonization, and nutrient accumulation in corn plants in coastal reclamation areas (Krishnamoorthy et al., 2016).

Two isolates, CK-24 and CK-54, belong to the genus *Bacillus*. The isolate CK-24 can produce IAA and ammonia and grow on DF and nitrogen-free media. However, CK-54 can solubilize phosphate, show IAA, protease, and ammonia production, and grow on DF media. Like *Pseudomonas*, *Bacillus* is also a widely used PGPR because of its various PGP traits and serves as biocontrol. Different *Bacillus* species have been used worldwide as PGPR. *Bacillus* is biocompatible with other genera, like nitrogen fixer *Azospirillum* and *Azotobacter*, and is used as a co-inoculant in the consortia of bacteria (Kashyap et al., 2019). *B. subtilis* has been reported to suppress pathogens and mitigate biotic stress in plants (Hashem et al., 2019).

One isolate, CK-40, was identified as *Klebsiella.* This isolate produces the highest level of IAA with 41^±0.40^µg/mL. It also showed an ability to grow on DF media, fix nitrogen, and solubilize phosphate, ammonia, and catalase production. The whole genome sequence analysis and other studies of a PGPR *Klebsiella* sp. D5A isolate from tall fescue roots revealed this bacterium’s PGPR, salinity-tolerant, and alkalotolerant properties (Liu et al., 2016). Another study showed that inoculating maize plants in greenhouse conditions with PGPR from *Klebsiella* isolates enhanced vegetative growth, nitrogen fixation, and nitrogen mobilization (Kuan et al., 2016).

The isolate CK-55 was identified as *Sphingobium* sp. This isolate can fix nitrogen, have vigorous ACC deaminase activity, and produce ammonia and catalase. *Sphingobium* belongs to the class Alpha-proteobacteria and is well-known for its pollutant bioremediation and biodegradation activities (Takeuchi et al., 2001). *Sphingobium* sp. strain AEW4 showed growth promotion in beachgrass and other plants, and comparative genomics analysis of its genome revealed several plant growth-promoting characteristics (Boss et al., 2022). Another strain of *Sphingobium*- DI-6 isolated from the diazinon-contaminated soil showed the capability to metabolize it as a carbon source and can be a potential candidate for diazinon bioremediation (Wang et al., 2022). Thus, CK-55 isolate can be a potential candidate for bioremediation and plant growth-promoting activities.

Eight bacterial isolates of 17 isolates, CK-6, CK-22, CK41, CK-44, CK-47, CK-50, CK-53, and CK55, showed a significant increase in the shoot biomass of *A. thaliana* (**Figure 4**). All isolates except CK-55 were identified as *Pseudomonas.* The CK-55 isolate identified as *Sphingobium* sp. (**Table 1**). All isolates showed IAA, siderophore and catalase production, phosphate solubilization, and nitrogen fixation abilities. Isolate CK-50 and CK-53 showed all the PGP traits tested in this study (**Table 2**). CK-53 showed a very vigorous growth on skim milk agar. *Pseudomonas* is the most investigated genus for plant growth-promoting characteristics and harboring many traits (Nadeem et al., 2016). *Pseudomonas* is one of the strong phosphates solubilizers among PGPR, and the gene responsible for mineral phosphate solubilization has been cloned from *Pseudomonas* isolate *P. cepacia* (Babu-Khan et al., 1995; Rodríguez and Fraga, 1999). In an iron-deficient environment, the *Pseudomonas* produce siderophore, enhancing iron availability for plant uptake (Rachid and Bensoltane, 2005).

*Pseudomonas* strains also reported mitigating various plant stresses because of different stress-related plant growth-promoting traits. A study has shown that *P. fluorescens* helps pea plants survive well in drought due to the better ability to colonize in the roots than *P. putida.* (Arshad et al., 2008). In contrast, the production of antifreeze protein by *P. putida* GR12-2 helped protect the canola plant from chilling injury(5°C) (Sun et al., 1995). Several ACC-deaminase enzymes-producing *Pseudomonas* sp. have been reported (Gupta and Pandey, 2019; Pandey and Gupta, 2020; Khan and Singh, 2021). Besides, various studies reported the presence of nitrogenase gene/island in *Pseudomonas* and their nitrogen-fixing capabilities (Krotzky and Werner, 1987; Yan et al., 2008; Li et al., 2017). *Pseudomonades* are well known to produce extracellular proteases. Various studies reported the biocontrol activities of different *Pseudomonas* sp. against plant pathogens due to their protease activities (Siddiqui et al., 2005; Durairaj et al., 2017; Ghadamgahi et al., 2022).

Snowbrush ceanothus is an evergreen actinorhizal plant that can fix atmospheric nitrogen and tolerate drought and heat. It is used in low-water-use landscaping and contains several plant growth-promoting bacteria but is difficult to propagate. The inoculation of bulk soil from the native location of snowbrush ceanothus to the cutting propagation mix promoted the cutting’s survival rate. The rhizobacteria isolated from these cuttings showed a high IAA production level and various plant growth-promoting characteristics like nitrogen fixation, phosphate solubilization, catalase, ammonia, and siderophore production, protease, and ACC deaminase activities. 50% of the isolates belong to *Pseudomonas*, and the other isolates identified as *Bacillus, Chryseobacterium, Massilia, Klebsiella*, and *Sphingobium* sp. The inoculation of eight bacterial isolates, CK-6, CK-22, CK41, CK-44, CK-47, CK-50, CK-53, and CK55, significantly increase the shoot biomass of *A. thaliana.* All except one CK-55 belong to *Pseudomonas*, CK-55 identified as *Sphingobium* sp. In this study, two of them, CK-50 and CK-53, exhibit all eight PGP traits. We are investigating these eight PGPR on maize, tall fescue, watermelon, and wheat for growth promotion under abiotic stresses. We are also testing these microbes for the cutting propagation of snowbrush ceanothus.

## Data availability statement

The datasets presented in this study can be found in online repositories. The names of the repository/repositories and accession number(s) can be found in the article/**Supplementary Material**.

## Author contributions

JG: Writing – review & editing, Validation, Methodology, Investigation, Formal analysis, Data curation. KH: Writing – review & editing, Validation, Methodology, Investigation, Formal analysis, Data curation. AD: Writing – review & editing, Validation, Methodology, Formal analysis. TW: Writing – review & editing, Methodology, Data curation. AK: Writing – review & editing, Writing – original draft, Visualization, Validation, Supervision, Software, Resources, Project administration, Methodology, Investigation, Funding acquisition, Formal analysis, Data curation, Conceptualization.
