# Supplementary material for: IAA-producing plant growth promoting rhizobacteria from Ceanothus velutinus enhance cutting propagation efficiency and Arabidopsis biomass
Source: Front Plant Sci. 2024 May 14;15:1374877. doi: 10.3389/fpls.2024.1374877 (PMC11131947; doi:10.3389/fpls.2024.1374877)
Supplement: Supplementary file 1 [file DataSheet_1.pdf]

Supplementary Figure 1

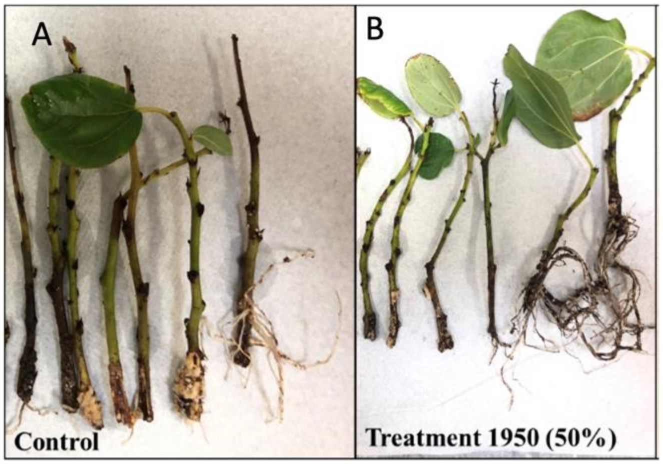

Supplementary Figure 1. Callus formation and rooting in snowbrush *ceanothus* cuttings. A) Control – Without native soil B) 50% Native soil from 1950.

Supplementary Figure 2

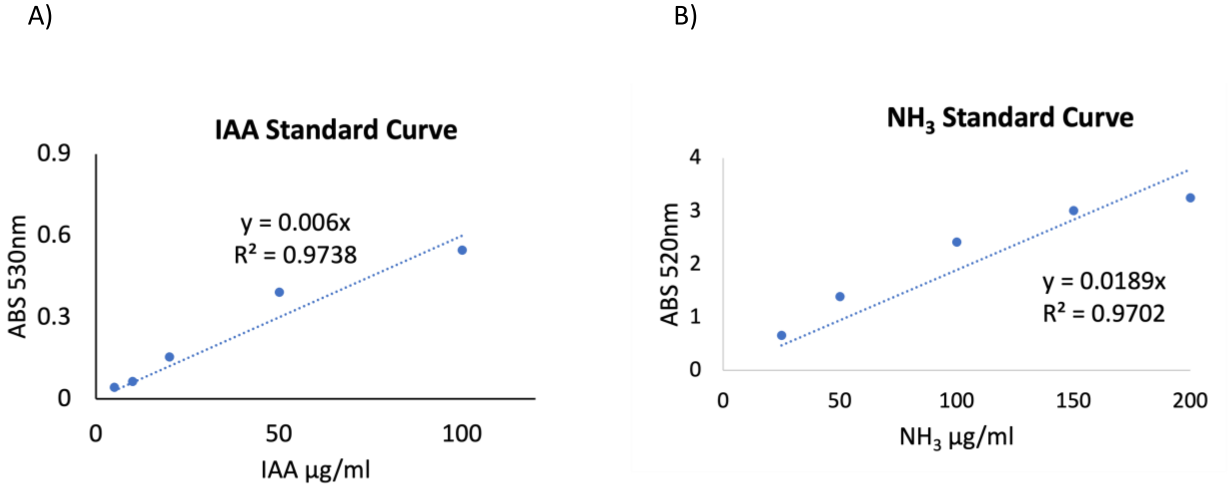

Supplementary Figure 2. Standard curve for A) IAA, B) Ammonia

Supplementary Table 1. Morphological characterization of seventeen isolates

S. No.	Code	Color	Texture	Transparency	Spread	Consistency	Morphology
1	CK-1	Mustard Yellow	Glossy	Translucent	Medium growth, dots	Viscous	No edges
2	CK-3	Cream	Glossy	Opaque/Trans lucent	Slow growth, thin	Viscous	Wavy translucent edges
3	CK-6	White	Matte	Translucent	Medium growth, thin	Viscous	Translucent edges
4	CK-20	Yellow	Glossy	Translucent	Fast growth, patchy	Dry	Sporadic Translucent edges
5	CK-22	White	Glossy/M atte	Translucent	Medium growth, dots	Viscous	Translucent edges
6	CK-24	White	Matte	Opaque	Medium growth, patchy	Dry	Translucent edges, faded at the tips
7	CK-25	Cream	Glossy	Opaque	Medium growth, thin	Viscous	No Edges
8	CK-29	Tan	Glossy	Translucent	Medium growth, dots	Viscous	No Edges
9	CK-40	White, yellow tint	Glossy	Translucent	Medium growth, dots	Viscous	Edges look like dried scales
10	CK-41	White	Glossy	Translucent	Medium growth, dots	Viscous	Edges even and Viscous
11	CK-44	White	Glossy	Translucent	Medium growth, dots	Viscous	Edges have fuzzy outline/fade
12	CK-47	White, pink tint	Glossy	Translucent	Medium growth, dots	Viscous	Translucent edges
13	CK-50	Cream	Glossy	Translucent	Medium growth, dots	Viscous/Dry	Transparent edges
14	CK-52	White	Glossy	Translucent	Medium growth, dots	Viscous	Translucent edges, fuzzy tips

15	CK-53	White & CLEAR	Glossy	Transparent	Medium growth, wide	Viscous	Viscous edges
16	CK-54	White	Glossy	Opaque	Fast growth, wide, wispy	Viscous	Translucent edges with extending lines with dots on end
17	CK-55	Bright White	Glossy	Translucent	Medium growth, dots	Viscous	Edges lighter yellow and translucent/opaque

Supplementary Table 2. IAA production in 27 isolates.

**Isolates**	**IAA (µg/mL)**
CK -1	$27.69^{\pm 1.73}$
CK - 3	$27.76^{\pm 0.58}$
CK - 4	$05.12^{\pm 0.31}$
CK - 5	$04.21^{\pm 0.20}$
CK - 6	$10.95^{\pm 0.02}$
CK - 7	$00.39^{\pm 0.21}$
CK - 11	$00.09^{\pm 0.08}$
CK - 13	$07.46^{\pm 0.05}$
CK - 14	$07.78^{\pm 0.26}$
CK - 20	$32.09^{\pm 0.30}$
CK - 22	$23.78^{\pm 0.36}$
CK - 24	$16.09^{\pm 0.72}$
CK - 25	$11.79^{\pm 0.12}$
CK - 27	$05.44^{\pm 0.03}$
CK - 29	$23.04^{\pm 0.67}$
CK - 31	$07.31^{\pm 0.06}$

CK - 35	$07.93^{\pm 0.15}$
CK - 40	$41.06^{\pm 0.40}$
CK - 41	$18.68^{\pm 0.59}$
CK - 43	$03.34^{\pm 0.03}$
CK - 44	$28.79^{\pm 0.54}$
CK - 47	$19.17^{\pm 0.20}$
CK - 50	$10.31^{\pm 0.23}$
CK - 52	$29.11^{\pm 0.42}$
CK - 53	$19.53^{\pm 0.18}$
CK - 54	$18.25^{\pm 0.11}$
CK - 55	$15.43^{\pm 0.12}$
